# Which one is the superior target? A comparison and pooled analysis between posterior subthalamic area and ventral intermediate nucleus deep brain stimulation for essential tremor

**DOI:** 10.1111/cns.13878

**Published:** 2022-06-10

**Authors:** Houyou Fan, Yutong Bai, Zixiao Yin, Qi An, Yichen Xu, Yuan Gao, Fangang Meng, Jianguo Zhang

**Affiliations:** ^1^ Department of Neurosurgery, Beijing Tiantan Hospital Capital Medical University Beijing China; ^2^ Beijing Key Laboratory of Neurostimulation Beijing China

**Keywords:** deep brain stimulation, essential tremor, posterior subthalamic area, secondary analysis, ventral intermediate nucleus

## Abstract

**Background/Aims:**

The efficacy and safety of posterior subthalamic area (PSA) and ventral intermediate nucleus (VIM) deep brain stimulation (DBS) in the treatment of essential tremor (ET) have not been compared in large‐scale studies. We conducted a secondary analysis to identify the superior target of ET‐DBS treatment.

**Methods:**

PubMed, Embase, Cochrane Library, and Google Scholar were searched for relevant studies before September 2021. The tremor‐suppression efficacy and rate of stimulation‐related complications (SRCR) after PSA‐DBS and VIM‐DBS treating ET were quantitatively compared. Secondary outcomes, including tremor subitem scores and quality of life results, were also analyzed. Subgroup analyses were further conducted to stratify by follow‐up (FU) periods and stimulation lateralities. This study was registered in Open Science Framework (DOI: 10.17605/OSF.IO/7VJQ8).

**Results:**

A total of 23 studies including 122 PSA‐DBS patients and 326 VIM‐DBS patients were analyzed. The average follow‐up time was 12.81 and 14.66 months, respectively. For the percentage improvement of total tremor rating scale (TRS) scores, PSA‐DBS was significantly higher, when compared to VIM‐DBS in the sensitivity analysis (*p* = 0.030) and main analysis (*p* = 0.043). The SRCR after VIM‐DBS was higher than that of PSA‐DBS (*p* = 0.022), and bilateral PSA‐DBS was significantly superior to both bilateral and unilateral VIM‐DBS (*p* = 0.001).

**Conclusions:**

This study provided level IIIa evidence that PSA‐DBS was more effective and safer for ET than VIM‐DBS in 12–24 months, although both PSA‐DBS and VIM‐DBS were effective in suppressing tremor in ET patients. Further prospective large‐scale randomized clinical trials are warranted in the future.

## Graphical abstract

This study provides level IIIa evidence that PSA‐DBS was more effective and safer for ET than VIM‐DBS in 12–24 months, though both PSA‐DBS and VIM‐DBS are effective in suppressing tremor in ET patients.

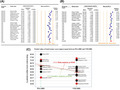

## 1 INTRODUCTION

Essential tremor (ET) is one of the most common movement disorders, with a prevalence of 0.9% worldwide and up to 5% in patients >65 years of age.^1^, ^2^ The pathophysiological mechanism remains uncertain, although several possible hypotheses have been proposed.^3^, ^4^ For drug‐resistant ET patients, deep brain stimulation (DBS) has been reported as a very useful surgical treatment. The thalamic ventral intermediate nucleus (VIM) is the most used target for ET‐DBS treatment, and VIM‐DBS is widely recognized to significantly improve ET symptoms.^5^, ^6^ However, the VIM target is difficult to visualize by conventional magnetic resonance imaging (MRI), adding extra resistances to target positioning. Although the patients' initial responses are usually encouraging, long‐term responses significantly decline. Furthermore, VIM‐DBS easily results in stimulation‐related complications (SRCs), such as dysarthria, gait ataxia, paresthesia, nausea, weakness, and other side effects.^5^, ^6^, ^7^

To solve these problems, the posterior subthalamic area (PSA), including the caudal zona incerta, Forel field H, and the prelemniscal radiation, was proposed as an effective and alternative stimulation target.^8^, ^9^, ^10^ Several studies proposed that PSA‐DBS might have better efficacy in controlling tremor symptoms and cause fewer SRCs,^8^, ^9^, ^10^, ^11^ and the implantation difficulty of PSA was much lower. Interestingly, it was even reported that PSA‐DBS was still effective in patients with failed VIM‐DBS.^8^ Although several reports compared the PSA and VIM stimulation for ET, the opinions were diverse and the outcomes were unconvincing, due to the retrospective design and the limited number of patients.^12^, ^13^, ^14^, ^15^, ^16^ Therefore, there is still controversy regarding the superior stimulation target for ET‐DBS treatment.

Here, VIM‐DBS and PSA‐DBS were compared with regard to the improvements in clinical symptoms and stimulation‐related complications based on published reports. The primary objective of our study was to identify the superior target of deep brain stimulation for essential tremor.

## 2 MATERIALS AND METHODS

### 2.1 Search strategy

The following databases were searched following the Preferred Reporting Items for Systematic Reviews and Meta‐Analyses (PRISMA) guideline^17^: PubMed, Embase, Cochrane Movement Disorders Group Trials Register, and Cochrane Central Register of Controlled Trials. Google Scholar was also searched for cited articles in references. The final search was in September 2021. The following keywords were used: “essential tremor,” “deep brain stimulation,” “ventral intermediate nucleus,” “posterior subthalamic area,” “caudal zona incerta,” “tremor scores,” “activities of daily living,” “complications,” and “side effects.” The titles, abstracts, full texts, and references were independently read and assessed by two investigators (FHY and BYT). Disagreements were settled through negotiations under the direction of ZJG. This study has already been registered in Open Science Framework (DOI: 10.17605/OSF.IO/7VJQ8).

### 2.2 Eligibility criteria and quality assessment

The inclusion criteria for included articles were: (1) the study reported PSA‐DBS and VIM‐DBS treatments for ET, (2) the study used objective scales including the Essential Tremor Rating Scale (ETRS) and/or the Fahn‐Tolosa‐Marin Tremor Rating Scale (FTM‐TRS) scores to report clinical outcomes, (3) the studies recorded the number of SRCs, (4) the study reported DBS targets, age at surgery, disease duration, total TRS scores both at baseline and the last follow‐up or postoperative percentage improvement, and (5) the follow‐up duration was longer than 3 months.

The exclusion criteria for eligible studies were: (1) indications for surgery other than ET, (2) a target other than PSA or VIM, (3) re‐implantation after failed DBS, (4) reports that included data that could not be extracted, (5) conference articles, (6) editorials, (7) reviews, (8) duplicate publications, (9) non‐English articles, and (10) low‐quality studies.

The quality of included studies was assessed by the Meta‐analysis of Observational Studies in Epidemiology (MOOSE) (Appendix S1).^18^, ^19^ A study was recognized as low quality when its MOOSE score was ≤3 points.

### 2.3 Data extraction

The data were extracted using a standardized template. The following items were collected: (1) characters of each article (number of ET patients, year of publication, journal of publication, and type of research); (2) baseline characteristics of the patients (sex, age at surgery, and disease duration); (3) surgical parameters, including stimulation targets (PSA or VIM), laterality of DBS (unilateral, bilateral, or both), and programming parameters; and (4) clinical outcomes evaluated when the patients were under individual medication treatments with the “on” state stimulation (ETRS or FTM‐TRS scores, follow‐up duration, stimulation‐related complications, and other scale scores at baseline and the last follow‐up). Because we wanted to compare the differences between PSA and VIM stimulation targets, adverse events related to DBS surgery and devices were excluded, and only stimulation‐related complications were included in the statistical analyses. Discrepancies were resolved by consultations between the authors (FHY and BYT).

### 2.4 Statistical analysis

Due to the heterogeneity of the TRS scales, no direct comparison could be made by the mean difference between the PSA‐DBS and VIM‐DBS groups. Hence, the effect size was determined by calculating the percentage improvements in tremor rating scale scores.^20^, ^21^ The percentage improvement was calculated as [(the presurgical score ‐ the postsurgical score)/the presurgical score × 100%]. The standard deviation (SD) was calculated as [(the SD of the presurgical score)^2^ + (the SD of the postsurgical score)^2^ – 2 × 0.6 × (the SD of the presurgical score) × (the SD of the postsurgical score)].^21^

The Standard Cochrane Q and I^2^ statistics were used to assess the heterogeneity. If *p* < 0.10 or I^2^ > 50%, the data were pooled by a random effect analysis model using a generic‐inverse variance. Otherwise, a fixed‐effect model was used. The mean ± standard error was used as a form of pooled data. Comparisons of patient baseline characteristics between the PSA and VIM groups were determined using Student's *t*‐tests. Comparisons of the main outcomes of the two groups, including the surgical effects and the rate of stimulation‐related complications (SRCR), were also performed using Student's *t*‐test. A value of *p* < 0.05 indicated a statistically significant difference. To estimate the study variance, a simple linear meta‐regression based on the method of the moment model was performed, and *p* < 0.05 was considered a significant correlation. Comprehensive Meta‐Analysis 3.0 (Biostat, Englewood, NJ, USA) was used to perform the statistical analyses. The data were managed using the MOOSE Group and the Cochrane Handbook for Systematic Reviews of Interventions.^19^

The average follow‐up time of PSA‐DBS and VIM‐DBS was 12.81 and 14.66 months, respectively. Therefore, we set a limitation of follow‐up (FU) time (12–24 months) to characterize middle‐term efficacies as the main analysis. Sensitivity analyses of clinical efficacy were conducted by considering all studies without limitation of FU time. Because all FU periods in the PSA‐DBS group were ≥12 months, a time limitation (≥12 months) was also set to determine the medium and long efficacy. The main and sensitivity analyses were also conducted with other assessment scales, including sub‐tremor scores of TRS, sub‐rest scores of TRS, sub‐upper extremities scores of TRS, sub‐functional disability of TRS, activities of daily life (ADL), short form‐36 (SF‐36), and quality of life (QoL) in the essential tremor questionnaire (QUEST). Subgroup analyses were further conducted to stratify follow‐up periods and stimulation lateralities.

Since most of the stimulation‐related complications (SRCs) would be alleviated or disappeared after adjusting the stimulation parameters, SRCs were not reported in some centers. So, the main analyses of SRCs were conducted without including studies that reported ‘0’ SRCs. For sensitivity analyses, all studies that reported the event number of SRCs were considered (Figure S1 **)**. Some common SRCs, including dysarthria and ataxia, were further independently analyzed. The rate of SRCs (SRCR) was calculated as [(the number of patients who occurred dysarthria) + (the number of patients who occurred ataxia) + … + (the number of patients who occurred one type of SRCs)/the total number of patients × 100%]. For example, if 6 of 10 patients occurred dysarthria and 5 of them occurred headache after stimulation, the SRCR would be (6 + 5)/10 × 100% = 110%.

## 3 RESULTS

### 3.1 Searching the results and quality assessments

**FIGURE 1:**
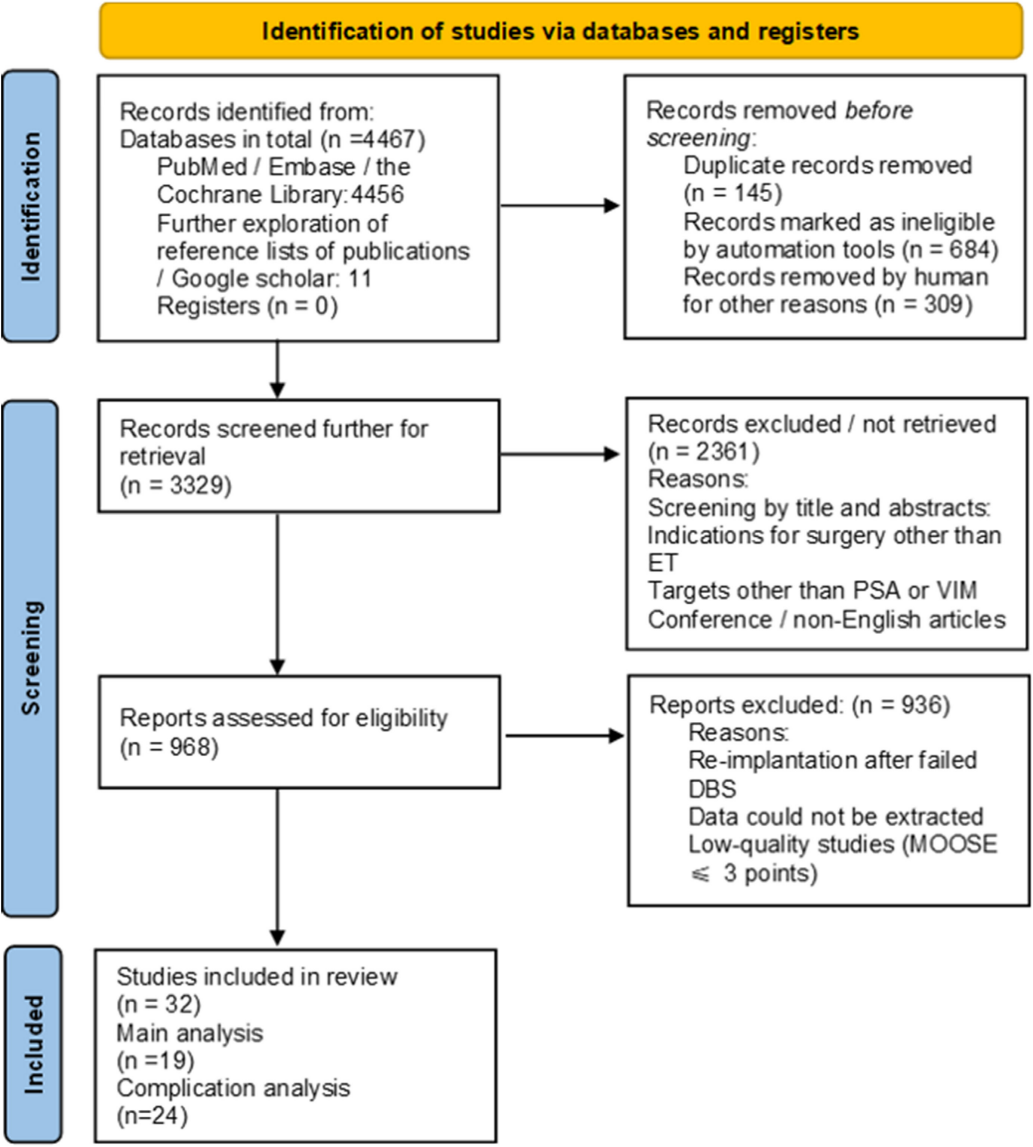
Preferred Reporting Items for Systematic Reviews and Meta‐Analyses 2020 flow diagram of studies included in the main and complication analyses (http://www.prisma‐statement.org/PRISMAStatement/FlowDiagram)

According to the keyword search, 4456 articles were identified, with a total of 1138 duplicate articles removed. The titles and abstracts were then filtered, excluding 2361 articles. The reasons for exclusion were that they were non‐ET projects, nonclinical studies, or low‐quality articles (conference articles, letters, or editorials). According to the inclusion and exclusion criteria, the remaining 968 articles were further screened by reading the full texts. The references to these articles were also screened. Finally, 32 studies were used for MOOSE quality assessment, with 22 studies involving efficacy analysis and 24 studies involving SRC analysis. The entire screening process is illustrated in Figure 1.

### 3.2 Study characteristics

No randomized controlled trial study was processed to determine the efficacy of ET‐DBS treatment. The detailed characteristics of included studies are shown in Table 1. For sensitivity analysis, eight studies were included in the PSA‐DBS group^13^, ^22^, ^23^, ^24^, ^25^, ^26^, ^27^, ^28^ and fourteen studies were included in the VIM‐DBS group.^5^, ^6^, ^13^, ^29^, ^30^, ^31^, ^32^, ^33^, ^34^, ^35^, ^36^, ^37^, ^38^, ^39^ Due to the short term of follow‐up, three studies in the VIM‐DBS group were excluded from the main analysis. The nineteen studies were published between 2004 and 2021. The improvement percentages ranged from 45.90% to 82.54% in the PSA‐DBS group and 36.6% to 75.8% in the VIM‐DBS group.

**TABLE 1:** Characteristics of treatment in each study

Study name	MOOSE	*N*	Age (years)	Disease duration (years)	Stimulation target	Follow‐up (months)	Unil/bil	Voltage (V)	Pulse widths (μs)	Frequency (Hz)	Preoperative TRS scores	Postoperative TRS scores	% Improvement
Studies including patients treated with PSA‐DBS													
Plaha P, 2004^19^	4	4	66.8 ± 8.5	17.3 ± 10.3	PSA	12	Bil	1.8 ± 0.2	108.8 ± 14.4	170 ± 11.5	63 ± 15.1	11.0 ± 3.9	82.54
Plaha P, 2011^20^	5	15	65.4 ± 7.9	21.5 ± 13.5	PSA	31.7	Bil	2.8 ± 1.2	112.5 ± 31.1	150 ± 22.9	63.9 ± 16.2	16.7 ± 9.3	73.87
Fytagoridis, A, 2016^21^	5	50	63.5 ± 13.1	NS	PSA	12	42/8	2.1 ± 0.8	64.6 ± 15.6	160.6 ± 21.6	47.6 ± 14.6	19.3 ± 13.0	59.45
Barbe MT, 2018^9^	6	6	NS	NS	PSA	12	Bil	NS	NS	NS	47.4 ± 4.9	17.2 ± 4.9	63.71
Degeneffe A, 2018^22^	4	3	58.3 ± 10.5	14 ± 4.6	PSA	70	Bil	NS	NS	NS	61.0 ± 5.6	33.0 ± 7.8	45.90
Andreas N, 2019^23^	5	11	67.0 ± 14.0	NS	PSA	12	Bil	2.3 ± 0.7	60 ± 0	140.4 ± 22.9	47.2 ± 15.7	21.3 ± 10.7	54.87
Sun XY, 2020^24^	4	7	59.0 ± 21.0	20 ± 11	PSA	12	Bil	2.4 ± 0.4	61.4 ± 8.6	139.3 ± 12.1	50.0 ± 10.0	14.0 ± 7.0	72.00
Philipson J, 2021^25^	5	26	65.8 ± 6.66	30.6 ± 20.1	PSA	12	20/6	1.8 ± 0.2	108.8 ± 14.4	170 ± 11.5	46.32 ± 14.08	17.0 ± 9.48	63.30
Studies including patients treated with VIM‐DBS													
Pahwa R, 2001^26^	5	17	73.1 ± 5.0	13.5 ± 6.84	VIM	3.1	Unil	NS	NS	NS	61.6 ± 13.2	30.5 ± 10.8	50.5
Vesper J, 2004^27^	5	18	NS	NS	VIM	12	NS	NS	NS	NS	57.76 ± 11.22	22.0 ± 6.5	44.6
Wildenberg WP, 2006^28^	4	10	61.7 ± 11.8	NS	VIM	38.4	3/7	NS	NS	NS	47.3 ± 23.42	30 ± 16.5	36.6
Blomstedt P, 2007^29^	5	19	66 ± 11.1	29.6 ± 14.4	VIM	13	Unil	1.8 ± 0.7	68.0 ± 14.0	164.0 ± 15.0	57.6 ± 19.2	29.2 ± 14.2	49.3
Ellis TM, 2008^30^	4	5	66 ± 11.1	31.5 ± 20.6	VIM	14	Unil	NS	NS	NS	40.4 ± 4.41	16 ± 9.82	60.4
Graft‐Radford J, 2010^31^	5	31	66.4 ± 10.7	12.6 ± 6.6	VIM	6	22/9	2.7	102.0	151	58.2 ± 14.8	23 ± 12.6	60.5
Zahos PA, 2013^32^	4	7	66.6 ± 10.6	25 ± 10.5	VIM	10.1	3/4	NS	NS	NS	31.8 ± 13	7.7 ± 4.5	75.8
Higuchi M, 2015^33^	5	44	65.5 ± 10.3	22.3 ± 13.5	VIM	12	NS	NS	NS	NS	53.7 ± 13.7	31.1 ± 12.6	42.1
Rodríguez C, 2016^34^	5	14	61.0 ± 2.5	23.5 ± 17.8	VIM	12	3/11	2.1 ± 0.6	90.0 ± 15.0	130.0 ± 1.0	63.3 ± 9.9	16.8 ± 11.7	73.5
Klein J, 2017^35^	5	26	67.27 ± 8.92	24.82 ± 17.37	VIM	24	Bil	NS	NS	NS	49.82 ± 16.55	21.21 ± 14.86	57.4
Barbe MT, 2018^9^	6	13	58.9 ± 17.0	9.8 ± 2	VIM	12	Bil	NS	NS	NS	47.4 ± 7.9	23.8 ± 6	49.8
Akram H, 2018^36^	4	5	63.8 ± 10.2	37 ± 3.8	VIM	23.6	Unil	2.2 ± 0.3	60.0 ± 0	NS	81.6 ± 17.6	48 ± 17.9	41.2
Paschen S, 2019^6^	5	20	66.6 ± 1.8	28.9 ± 16.9	VIM	13.1	Bil	2.44 ± 0.2	66.0 ± 2.8	145.5 ± 5.5	56.3 ± 16.55	20.9 ± 11.77	62.9
Tsuboi, 2020^7^	5	97	67.4 ± 9.6	13.5 ± 6.84	VIM	12	72/25	2.7 ± 0.7	92.0 ± 25.7	92.0 ± 25.7	51.2 ± 14.8	22.9 ± 12.9	55.3

Abbreviations: DBS, deep brain stimulation; MOOSE, Meta‐analysis of Observational Studies in Epidemiology; NS, not specified; PSA, posterior subthalamic area; TRS, tremor rating scale; Unil/bil, unilateral/bilateral; VIM, ventral intermediate nucleus.

### 3.3 Baseline characteristics and stimulation parameters of PSA‐DBS and VIM‐DBS

The comparison results of baseline characteristics and stimulation parameters between PSA‐DBS and VIM‐DBS are summarized in Table 2. There was no significant difference between the PSA‐DBS and VIM‐DBS groups of the baseline characteristics, including age at surgery (64.69 ± 1.71 vs. 65.96 ± 1.09 years, *p* = 0.47), disease duration (20.66 ± 6.02 vs. 23.37 ± 3.99 months, *p* = 0.56), and length of follow‐up (12.81 ± 0.61 vs. 14.66 ± 2.06 months, *p* = 0.28). No statistical difference was found in frequency (152.21 ± 7.59 vs. 132.78 ± 8.13 Hz, *p* = 0.15), pulse width (78.47 ± 6.24 vs. 79.40 ± 5.45 μs, *p* = 0.99), or amplitude (2.20 ± 0.14 vs. 2.36 ± 0.11 V, *p* = 0.45). There was no significant difference in presurgical total TRS scores (52.53 ± 2.83 vs. 53.55 ± 2.12, *p* = 0.80), and the same statistical consequences were present in the baseline characteristics and stimulation parameters of the main analysis.

**TABLE 2:** Pooled value of demographics^a^

	PSA‐DBS	VIM‐DBS	*p*‐value
Age of surgery	64.69 ± 1.71 (116)	65.96 ± 1.09 (308)	0.47
Disease duration (years)	20.66 ± 6.02 (83)	23.37 ± 3.99 (248)	0.56
Follow‐up duration (months)	12.81 ± 0.61 (122)	14.66 ± 2.06 (326)	0.28
Frequency (Hz)	152.21 ± 7.59 (87)	132.78 ± 8.13 (150)	0.15
Pulse widths (μs)	78.47 ± 6.24 (87)	79.40 ± 5.45 (186)	0.99
Amplitude (V)	2.20 ± 0.14 (87)	2.36 ± 0.11 (186)	0.45
Preoperation total TRS	52.53 ± 2.83 (122)	53.55 ± 2.12 (326)	0.80
Improvement rate of total TRS			
Sensitivity analysis (no time limitation)	64.89 ± 3.14 (122)	56.23 ± 2.44 (326)	**0.030**
Sensitivity analysis^b^(≥12 M)	64.89 ± 3.14 (122)	54.44 ± 2.75 (271)	**0.012**
Main analysis (12 M‐24 M)	66.11 ± 4.09 (104)	55.56 ± 3.24 (261)	**0.043**
SRCR (%)	48.2 ± 22.1 (89)	106.3 ± 12.4 (516)	**0.022**
Rate of dysarthria (%)	29.2 ± 10.6 (86)	25.5 ± 6.1 (485)	0.147

Abbreviations: DBS, deep brain stimulation; PSA, posterior subthalamic area; SRCR, rate of stimulation‐related complications; TRS, tremor rating scale; VIM, ventral intermediate nucleus.

^a^ “Mean ± standard error (number of observations)” is used to represent the data.

^b^ The sensitivity analysis with a limitation of the follow‐up time (≥12 months).

*p*‐values of comparisons with significant differences are highlighted in bold.

### 3.4 Outcomes of DBS efficacy

**FIGURE 2:**
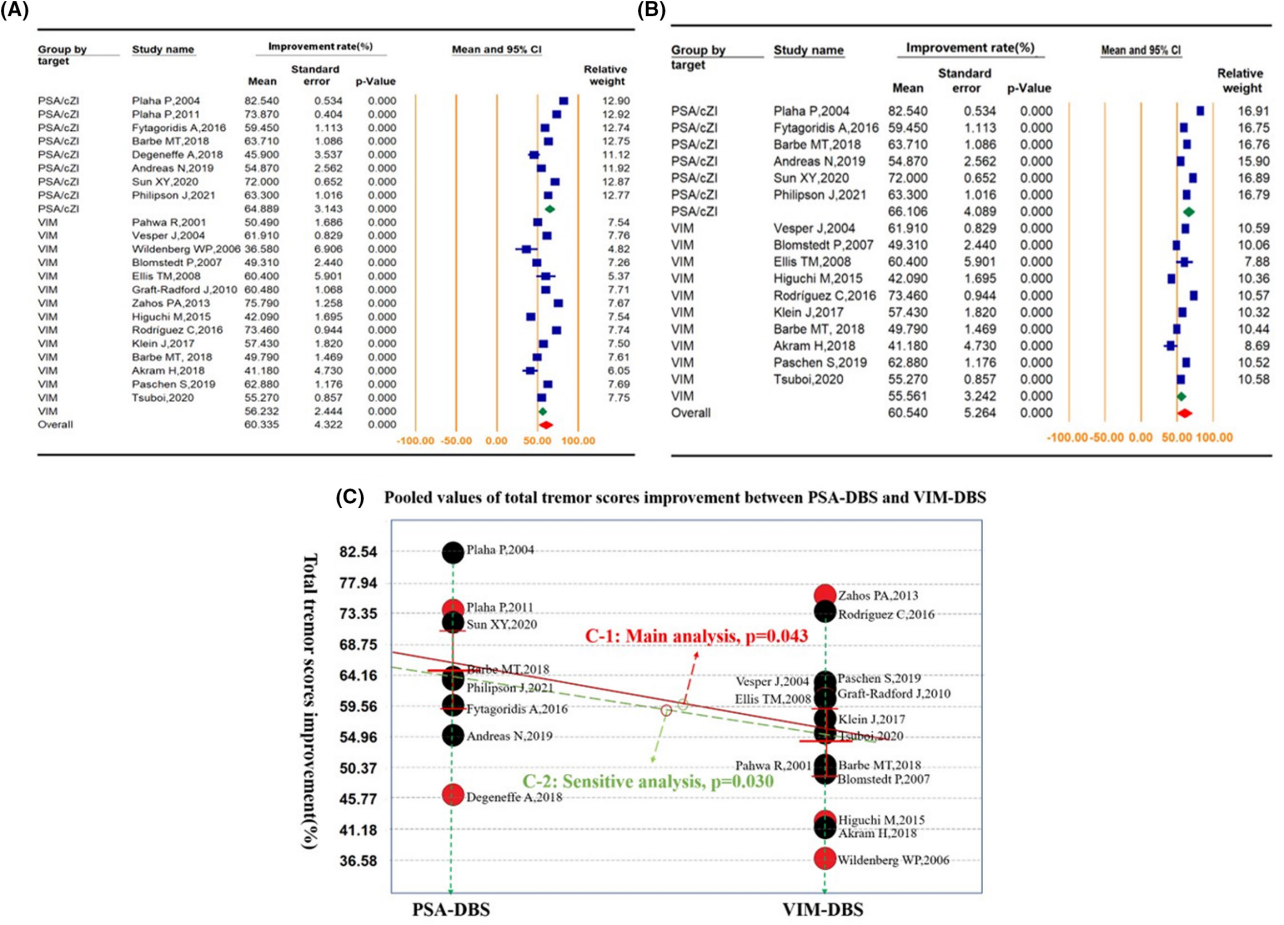
Differences of total tremor score improvement (%) between PSA and VIM deep brain stimulation. (A) The forest plot of sensitivity analysis with no time limitations of the follow‐up periods. (B) The forest plot of main analysis with the follow‐up period limited to 12–24 months. (C) The *p*‐value of main analysis (*p* = 0.043, 12 − 24 M, C‐1) and sensitivity analysis (*p* = 0.030, no time limitation, C‐2). PSA—posterior subthalamic area; VIM—ventral intermediate nucleus; DBS—deep brain stimulation

The improvement of total TRS scores was used as the primary evaluation indicator. Statistical differences were found between the PSA‐DBS and VIM‐DBS groups in the sensitivity analysis with no follow‐up time limitation (64.89 ± 3.14% vs. 56.23 ± 2.44%, *p* = 0.030) and main analysis with a time limitation of 12–24 months (66.11 ± 4.09% vs. 55.56 ± 3.24%, *p* = 0.043). Significant differences were also found during further analysis of medium and long efficacy (64.89 ± 3.14% vs. 54.44 ± 2.75%, *p* = 0.012). The forest plots and comparison outcomes are shown in Figures 2 and S2. We also analyzed the comparison outcomes of the sub‐action, rest, midline, and extremity tremor scores of TRS, sub‐functional disability of TRS, activities of daily life (ADL), short form‐36 (SF‐36), and QoL in the essential tremor questionnaire (QUEST). For the percentage improvement of total TRS scores, the bilateral PSA‐DBS was significantly higher with no FU‐time limitation (*p* = 0.066) and significantly higher with an FU‐time limitation (12–36 months, *p* = 0.001), when compared to the bilateral VIM‐DBS. The bilateral PSA‐DBS was also significantly higher than the unilateral VIM‐DBS (*p* = 0.001). However, no statistical difference was found between the bilateral and unilateral VIM‐DBS (*p* = 0.192). These statistical consequences were of limited value due to insufficient data, especially for subgroups stratified by follow‐up periods, where only one study of PSA‐DBS was followed for more than 3 years.

### 3.5 Outcomes of stimulation‐related complications

**FIGURE 3:**
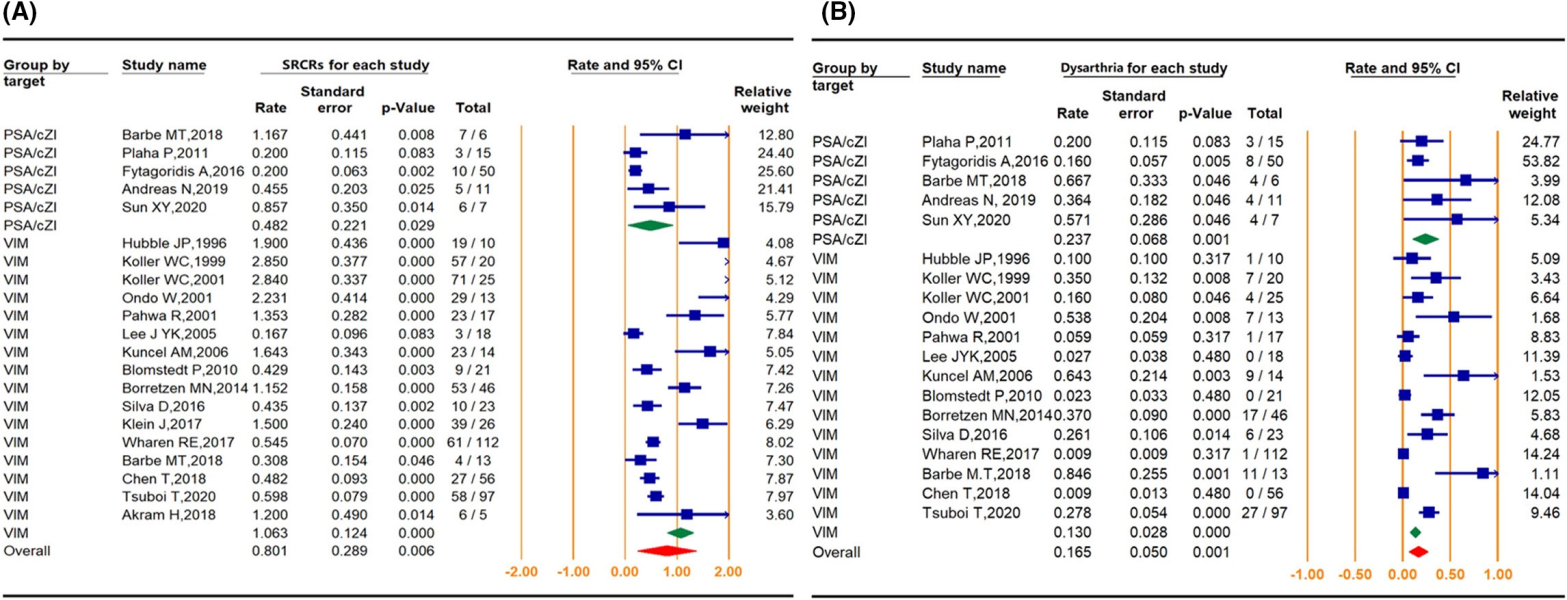
Forest plots of the SRCR (A) and dysarthria rate (B) between PSA and VIM deep brain stimulation. SRCR, rate of stimulation‐related complications; PSA, posterior subthalamic area; VIM, ventral intermediate nucleus

The summary of SRCs was also an important evaluation indicator. A total of 439 SRCs were reported in 479 ET patients treated with PSA‐DBS and VIM‐DBS^6^, ^9^, ^13^, ^22^, ^25^, ^26^, ^37^, ^38^, ^39^, ^40^, ^41^, ^42^, ^43^, ^44^, ^45^, ^46^, ^47^, ^48^, ^49^ (Table 3). The occurrence of SRCR of VIM‐DBS was statistically higher than that of PSA‐DBS (106.3 ± 12.4 vs. 48.2 ± 22.1%, *p* = 0.022). The occurrence of SRCs ranged from 20.0% to 116.7% in the PSA‐DBS group and 30.8% to 285.0% in the VIM‐DBS group, and there was no significant difference in the occurrence of dysarthria between the two groups (29.2 ± 10.6% vs. 25.5 ± 6.1%, *p* = 0.147). The occurrence of dysarthria ranged from 16.0% to 66.7% in the PSA‐DBS group and 10.0% to 84.6% in the VIM‐DBS group. The forest plots and comparison outcomes are shown in Figure 3. No significant difference was found in the occurrence of ataxia (Figure S3).

**TABLE 3:** Summary of stimulation‐related complications^a^

Study name	*N*	Stimulation‐related complications	Dysarthria	Ataxia	*n*
Studies including patients treated with PSA‐DBS					
Plaha P, 2011^20^	15	Bilateral dysarthria and a hypophonic (3)	0	0	3
Blomstedt P, 2010^37^	28	Dysarthria (8), clumsiness in the contralateral hand and leg (1), blurred vision and dizziness (1)	3	0	10
Barbe MT, 2018^9^	6	Dysarthria (4), gait ataxia (3)	8	0	7
Andreas N, 2019^23^	11	Mild dysarthria (4), paresthesia (1)	4	3	5
Sun XY, 2020^24^	7	Mild dysarthria (4), mild balance disorder (2)	4	0	6
Studies including patients treated with VIM‐DBS					
Hubble JP, 1996^38^	10	Paresthesia (10), dysarthria (1), headache (2), face‐arm pain (1), right‐sided weakness (3), face weakness (1), decreased range of motion left shoulder (1)	1	0	19
Koller WC, 1999^39^	20	Mild paresthesia (24), mild headache (9), mild dysarthria (7), mild paresis (6), attention/cognitive deficits (2), gait disorder (2), facial weakness (2), dystonia (1), hypophonia (1), nausea (1), mild depression (1), dizziness (1)	7	2	57
Koller WC, 2001^40^	25	Paresthesia (21), headache (15), paresis (6), dysarthria (4), nausea (4), disequilibrium (3), facial weakness (3), gait disorder (2), dystonia (2), mild attention/cognitive deficit (2), dizziness (2), hypophonia (1), anxiety (1), depression (1), syncope (1), vomiting (1), shocking sensation (1), drooling (1)	4	2	71
Ondo W, 2001^41^	13	Paresthesia (3), headache (5), dysarthria (7), neck pain (2),mouth pain (1),increased saliva (1), balance and gait difficult (10)	7	10	29
Pahwa R, 2001^26^	17	Headache (9), paresthesia (10), dysarthria (1), disequilibrium (1), dizziness (2)	1	1	23
Lee JYK, 2005^42^	18	Hand tingling (3)	0	0	3
Kuncel AM, 2006^43^	14	Dysarthria (9), posturing (7), jaw deviation (3), eye closure (2), voice effected (2)	9	7	23
Blomstedt P, 2010^37^	21	Aphasia (8), clumsy (1)	0	0	9
Borretzen MN, 2014^44^	46	Dysarthria (17), headache (9), paresthesia (6), abnormal taste (8), dizziness (5), discomfort tongue (4), reduced balance or coordination (4)	17	4	53
Silva D, 2016^45^	23	Paresis (2), dysarthria (6), transient cognitive alter (1), facial numbness (1)	6	0	10
Klein J, 2017^35^	26	Dysarthria (15), gait/balance (11), paresthesia (8), dysphagia (2), increased headaches (1), dizziness (1), cramps (1)	15	11	39
Wharen RE, 2017^46^	112	Speech disturbances (12), gait/postural disorder (5), cognitive changes (8), dysphagia (2), tinnitus (1), shocking or Jolting sensation (3), discomfort (17), headache (8), paresis (1), dystonia (2), dysarthria (1), hemiparesis (1)	1	5	61
Barbe MT, 2018^9^	13	Right hemiparesis (1), dysarthria (11), aphasia (1), nausea (1)	11	0	4
Chen T, 2018^47^	56	Mental status change (9), speech disturbance (7), balance or gait disturbance (6), speech and balance disturbances (5)	0	6	27
Akram H, 2018^36^	5	Mild slurring and slowing of speech (1), tingling (1), discomfort in the right side of the face, right arm, and part of the right leg (1), mild balance deterioration (1), feeling of exhaustion (1), mild and transient paresthesia (1)	0	1	6
Tsuboi T, 2020^7^	97	Dysarthria (27), gait/postural disorders (19), dysphagia (6), paresthesia (2), limb ataxia (3), double vision (1)	27	19	58

Abbreviations: DBS, deep brain stimulation; *N*, number of patients; *n*, number of stimulation‐related complications; PSA, posterior subthalamic area; VIM, ventral intermediate nucleus.

^a^ Despite their different names, some studies in the stimulation‐related complications summary reported the same group of patients as those in the total tremor score improvement summary.

## 4 DISCUSSION

Drug treatment has long been the main therapy for ET, but only 50% of ET patients are sensitive to pharmacological treatment.^50^ Due to nonablative, adjustable, and reversible characteristics, DBS became an alternative and effective treatment for drug‐resistant ET patients. PSA and VIM are the two most common stimulation targets of ET‐DBS treatment.

In recent years, several reviews studying ET‐DBS treatments have been published,^2^, ^10^, ^20^ which characterized the efficacy and safety of ET‐DBS treatments. However, none of the literature reports compared the differences between PSA‐DBS and VIM‐DBS.

Our study conducted deeper analyses of the effects of ET‐DBS treatment to obtain level IIIa evidence that PSA‐DBS was more effective and safer for ET than VIM‐DBS in 12–24 months, although both PSA‐DBS and VIM‐DBS were effective in suppressing tremor in ET patients.

### 4.1 The efficacy between PSA‐DBS and VIM‐DBS

Overall, ET patients treated with PSA‐DBS and VIM‐DBS both showed statistically significant improvements, and PSA‐DBS was superior to VIM‐DBS in terms of the total TRS scores. The clinical outcomes of PSA‐DBS and VIM‐DBS for ET patients have only been directly compared in six studies.^12^, ^13^, ^14^, ^15^, ^16^, ^24^ Although all of them concluded that PSA‐DBS and VIM‐DBS were both effective in the treatment of ET patients, the conclusions were still diverse. Barbe, Holslag, and Sandvik et al.^12^, ^13^, ^16^ all suggested that PSA was possibly a superior target in deep brain stimulation for essential tremor. Blomstedt and Degeneffe et al.^14^, ^24^ found no statistical difference in the reduction of total TRS scores between PSA‐DBS and VIM‐DBS. Eisinger et al.^15^ reported that VIM‐DBS provided better long‐term outcomes in terms of sub‐tremor scores. Our analysis supported the above opinion that PSA was the superior target. Compared with VIM‐DBS, total TRS scores were more improved after PSA‐DBS. Several potential reasons contributed to this result.

According to current theories, the generation of ET is attributed to multiple central oscillators across the cerebello‐thalamo‐cortical circuit (CTCC) dynamically attracted to each other to induce ET symptoms.^51^, ^52^ In theory, interfering with any node of this circuit could suppress tremor oscillations. This may be due to the inhibition of cerebellum‐cortex connections by local DBS, or to more complex mechanisms such as multifocal alterations in efficient and functional connectivity throughout neural networks.^53^, ^54^ Tremor improvement has been reported to be significantly correlated with primary sensorimotor regions, supplementary motor areas (SMAs), and premotor cortex in ET.^55^, ^56^ VIM is in the center of the CTCC, which connects the primary motor cortex (M1) and the dentate nucleus of the contralateral cerebellum across the dental‐red‐thalamic tract (DRTT), projecting to the tremor‐related motor areas. Therefore, it has been considered an effective target for lesioning surgery and neuromodulation to suppress tremor symptoms.

However, in recent years, increasing interest has focused on PSA‐DBS for tremor control. VIM‐DBS was reported to achieve its best effects in the subthalamus region, which was adjacent to PSA. A positive correlation between tremor inhibition and stimulation of DRTT was shown by many imaging studies.^38^, ^57^, ^58^, ^59^ PSA was proposed to be more closely related to DRTT. Anatomically, the fibers originate from the dentate nucleus of the cerebellum and climb across the superior cerebellar peduncle into the caudal mesencephalon. Most of them then crisscross to attain the red nucleus and thalamus of the contralateral hemisphere. Smaller, noncrossing DRTT processes reach the red nucleus and thalamus of the ipsilateral hemisphere. The rising DRTT fibers need to transit the small and the narrow PSA, then spread and end in the thalamus. The proximity of DRTT fibers in PSA makes it a good target for DBS. Al‐Fatly et al. concluded that these targets might be the same fibers, which were transmitted to the thalamus along with the red nucleus and passed through the PSA and zona incerta during this process.^59^ An optimal DBS spot was proposed to be located outside the ventrolateral thalamus, inside the internal capsule, directly below the VIM and sensory nuclei of the thalamus, invading their inferior borders.^60^ Overall, the superior location of PSA may contribute to the better results of tremor suppression.

It has also been suggested that conventional MRI is unable to adequately visualize the VIM region. Though several specific (typically proton density) MRI sequences or tractography were reported to be able to visualize the VIM, they are not routinely used in most centers.^61^ Atlas‐defined coordinates have therefore been heavily used to indirectly localize the stimulation target. VIM is between ventro‐oralis posterior (VOP) and ventral caudal (VC), with a front and back diameter of only 3–4 mm, which is very small and narrow. If the location is inaccurate, it may not be in the VIM nucleus, especially if the Y value is uncertain, causing poor therapeutic results.^11^ PSA has connected anteriorly to the posterior edge of the STN, posteriorly to the anterolateral edge of red nucleus, and laterally to the posterior limb of the inner capsule.^9^ Compared with the VIM nucleus, the difficulty in locating the PSA is much lower. Therefore, the therapeutic effect of PSA‐DBS is more controllable, while VIM‐DBS may fail to achieve the best therapeutic effects due to inaccurate target positioning.

The efficacy of bilateral PSA‐DBS was significantly better than that of bilateral and unilateral VIM‐DBS. Notably, no statistical difference was found between bilateral and unilateral VIM‐DBS. Many studies have also come to similar conclusions that the bilateral and unilateral VIM‐DBS were equally effective for ET.^62^, ^63^ Nevertheless, we must cautiously consider the results of this subgroup analysis. Only a limited number of articles have reported unilateral and bilateral stimulations on all included ET patients, and many of them involved mixed targets.

Regarding long‐term efficacy, PSA and VIM stimulation both significantly decreased with longer follow‐up periods. This may be due to the increased tolerance of patients to stimulation, and the progressing process of ET.^31^, ^64^ Disease progression was also recognized as an important reason for the loss of efficacy.^65^ Anthofer et al. reported that patients with long distant contact with DRTT fibers were more prone to suffer from DBS tolerance.^66^ PSA‐DBS may have a better long‐term efficacy due to its closer location to the DRTT fibers when compared to VIM‐DBS.^67^

According to multiple reports, VIM‐DBS improves the QoL in ET patients. However, only five studies reported the QoL of PSA‐DBS using three different types of assessment scales, including SF‐36, ADL, and QUEST.^13^, ^22^, ^24^, ^27^, ^28^ It is of limited value to use such diverse data to make a comparison with VIM‐DBS. But according to their reports, both PSA‐DBS and VIM‐DBS could improve the QoL for ET patients. No significant difference was observed in terms of the percentage improvement in ADL.^14^

### 4.2 The stimulation‐related complications between PSA‐DBS and VIM‐DBS

Although many studies have mentioned adverse effects, they were limited to complications related to surgery and equipment, which were of a diverse nature due to the medical level of different centers. SRCs are side effects directly related to or introduced by DBS, which are more important in judging the safety of different stimulation targets. These adverse reactions may or may not disappear with the adjustment of parameters, so any adverse events that emerge should be included in this analysis. In our analysis, the pooled percentages of SRCR after PSA‐DBS were significantly lower than that after VIM‐DBS. Holslag and Barbe et al. also reported a similar conclusion.^13^, ^16^ Different anatomical locations have been recognized as the key factors contributing to different SRCR.^38^, ^57^, ^58^, ^59^

Dysarthria and gait ataxia have been reported as two of the most common SRCs in DBS. The occurrences of dysarthria and gait ataxia were diverse in different studies, but we found that they were common and similar in both PSA‐DBS and VIM‐DBS.^10^, ^16^ It has been proposed that dysarthria and gait ataxia are caused by stimulation of the same cerebellar fiber connections throughout the cerebello‐thalamo‐cortical circuit in both PSA‐DBS and VIM‐DBS, including afferent or efferent axons of the red nucleus and other adjacent tracts, which contributed to similar occurrence percentages.^68^, ^69^, ^70^

### 4.3 The stimulation parameters between PSA‐DBS and VIM‐DBS

With different programming parameters, the efficacy and SRCs of PSA‐DBS and VIM‐DBS will also be different. Therefore, all included reports in the literature have a uniform inclusion criterion, including data from the “ON state” and normal follow‐up periods, which ensured that the parameters are the timely parameters coordinated by the physicians and patients after considering individual efficacies and tolerabilities.

Stimulation parameters, including voltage, pulse width, and frequency are interdependent and should be considered together.^71^ There were no statistical differences in these stimulation parameters, which was the premise of our comparisons and analyses. Interestingly, ET‐DBS treatment was generally recognized to have a better therapeutic effect at high frequency, so the frequencies of PSA‐DBS and VIM‐DBS were both high.^71^, ^72^

To improve tremor symptoms, many patients were even willing to tolerate mild SRCs, such as mild dysarthria and gait ataxia.^72^, ^73^, ^74^ With the increasing understanding of DBS programming, adaptive and/or sensing closed‐loop DBS, delivering stimulation only when necessary to reduce SRCs and prolong clinical efficacy, is considered as a promising ET‐DBS treatment, when compared with conventional continuous DBS.^75^, ^76^ While conventional DBS electrode contacts stimulate the ring‐shaped area around the electrode, directional electrodes achieve stimulation in different directions by dividing the ring electrode into segmented electrodes. Based on these clinical characteristics, including local field potential (LFP), a larger treatment window and more precise stimulation can be achieved.^77^, ^78^

## 5 LIMITATIONS

### 5.1 Our study had several limitations

First, most included studies were one‐arm studies that did not contain the controlled trial design. However, more ET patient samples were included in this way, which improved the universality and statistical validity of our results. Furthermore, one‐arm meta‐analysis is also regarded as a reliable method to provide level IIIa evidence.^20^, ^21^, ^79^, ^80^

Second, because most of the follow‐up times in PSA‐DBS were in 12–24 months, and our outcomes mainly reflected medium–long‐term results, more studies are needed to better compare short‐term and long‐term efficacies.

Finally, non‐English studies were excluded, which reduced the number of relevant studies included in our analyses.

## 6 CONCLUSIONS

Our study demonstrated favorable outcomes in terms of clinical efficacy and safety. Although PSA‐DBS and VIM‐DBS were both effective for ET, the efficacy of PSA‐DBS in tremor suppressing was superior, and PSA‐DBS caused fewer stimulation‐related complications. Hence, this study provided level IIIa evidence that PSA‐DBS was more effective and safer for ET than VIM‐DBS in 12–24 months.

Considering the diverse and insufficient data of QoL, a gold standard assessment scale is required for future comparison. Outcomes of longer‐term follow‐ups or bilateral DBS should be emphasized and collected. Further prospective large‐scale randomized clinical trials are warranted in the future. Collectively, we believe that past, present, and future studies should enable clinicians to better understand the ET‐DBS treatment and make the optimal choices.

## AUTHOR CONTRIBUTION

Jianguo Zhang contributed to the concept and design of this manuscript. Houyou Fan and Yutong Bai collected the data and wrote the manuscript. Zixiao Yin and Fangang Meng helped the methodology and visualization. Qi An, Yichen Xu, and Yuan Gao joined the process of data curation and formal analysis.

## CONFLICTS OF INTEREST

No conflict of interest exists in this study.

## Supporting information


Appendix S1

## Data Availability

All data used to conduct analyses are available from the public published papers and the corresponding author upon reasonable request.
