# Supplementary material for: Which one is the superior target? A comparison and pooled analysis between posterior subthalamic area and ventral intermediate nucleus deep brain stimulation for essential tremor
Source: CNS Neurosci Ther. 2022 Jun 10;28(9):1380–92. doi: 10.1111/cns.13878 (PMC9344089; doi:10.1111/cns.13878)
Supplement: Supplementary file 1 — Appendix S1 [file CNS-28-1380-s001.docx]

***Details of the Meta-analysis of Observational Studies in Epidemiology (MOOSE)***

Each of the following items was equal to one point, with a maximum of six points: (1) a clear definition of the study population and an adequate number of patients (n > 10); (2) unambiguous definitions and assessments of outcomes; (3) independent evaluation of outcome parameters; (4) an unambiguous follow-up description; (5) no or little selective bias (<10%); and (6) identification of prognostic factors and significant confounders.


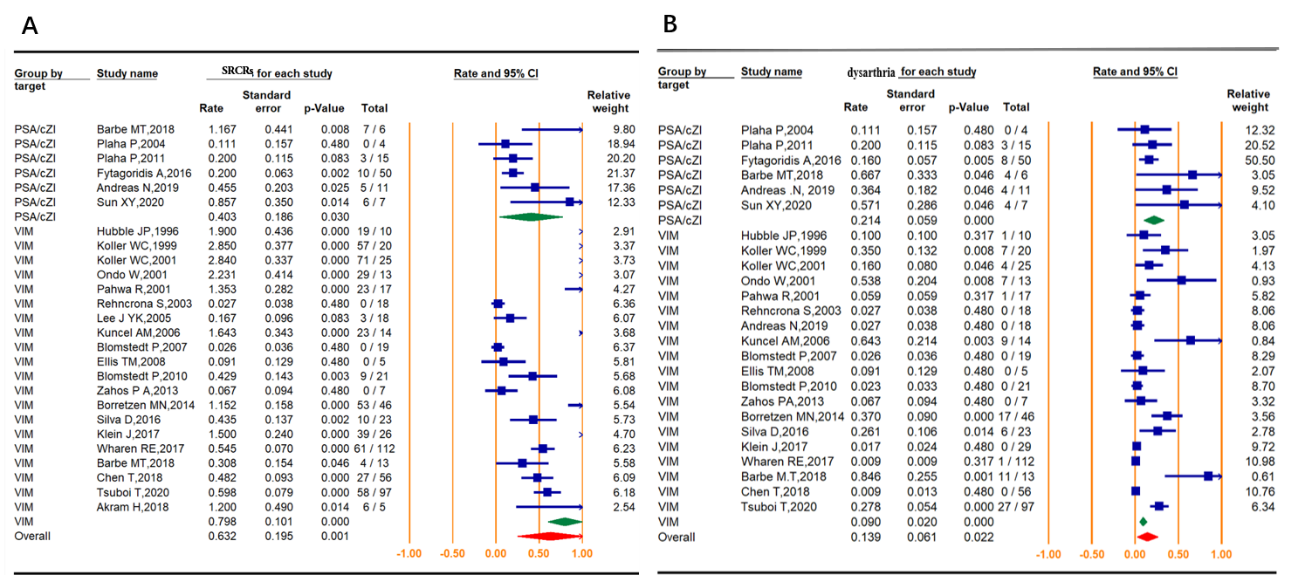


**FIGURE S1.** Forest plots of SRCRs(A) and dysarthria rates(B) between PSA and VIM deep brain stimulation in the sensitivity analysis. SRCRs: the rates of stimulation-related complication; PSA: posterior subthalamic area; VIM: ventral intermediate nucleus.


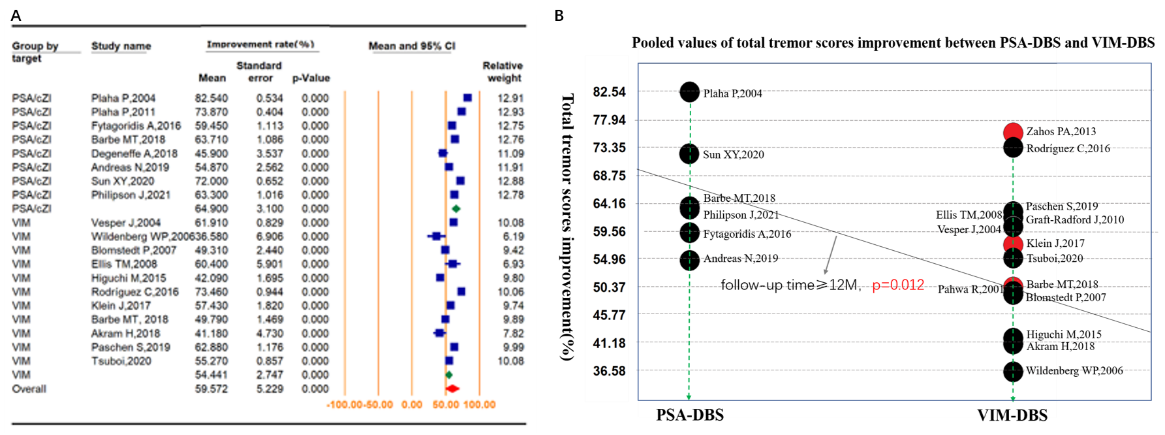


**FIGURE S2.** The differences of total tremor scores improvement(%) between PSA and VIM deep brain stimulation. **A:** The forest plot with the follow-up period limited to ≥12 months. **B:** p-value of medium and long efficacy(p=0.012. ≥12M). PSA: posterior subthalamic area; VIM: ventral intermediate nucleus; DBS: deep brain stimulation.


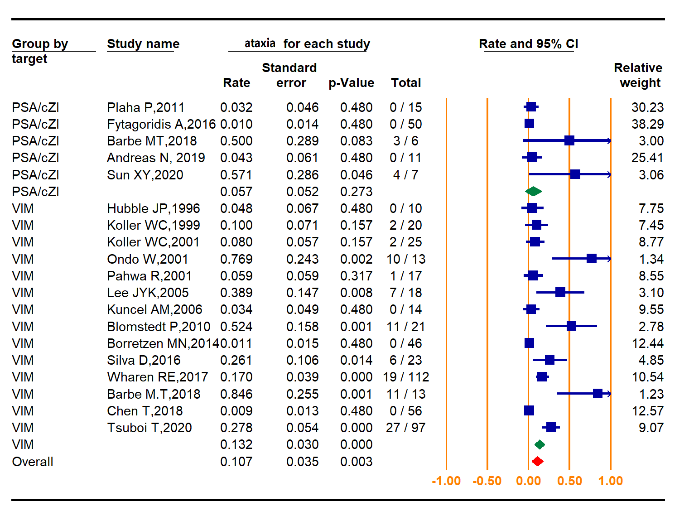


**FIGURE S3.** The forest plot of ataxia rates between PSA and VIM deep brain stimulation.
